# *Age Nutrition Chirugie* (ANC) study: impact of a geriatric intervention on the screening and management of undernutrition in elderly patients operated on for colon cancer, a stepped wedge controlled trial

**DOI:** 10.1186/s12877-016-0402-3

**Published:** 2017-01-07

**Authors:** Marine Dupuis, Elisabetta Kuczewski, Laurent Villeneuve, Sylvie Bin-Dorel, Max Haine, Claire Falandry, Thomas Gilbert, Guillaume Passot, Olivier Glehen, Marc Bonnefoy

**Affiliations:** 1 Unité de Recherche Clinique, Pôle Information Médicale Evaluation Recherche, Hospices Civils de Lyon, Lyon, France; 2 Service d’Hygiène Épidémiologie Infectiovigilance et Prévention, Hospices Civils de Lyon, Lyon, France; 3 Université de Lyon, EAM Parcours Santé Systémique, 4128 Lyon, France; 4 Université Lyon 1, Lyon, France; 5 Service de Médecine Gériatrique, Centre Hospitalier Lyon-Sud, Hospices Civils de Lyon, Pierre-Bénite, France; 6 Université Lyon 1, EMR 3738, Oullins, France; 7 Oncologie Médicale, Centre d’Investigation des Thérapeutiques en Oncologie et Hématologie de Lyon (CITOHL), Centre Hospitalier Lyon-Sud, Institut de Cancérologie des Hospices Civils de Lyon (IC-HCL), Lyon, France; 8 Service de Chirurgie Viscérale et Endocrinienne, Centre Hospitalier Lyon-Sud, Hospices Civils de Lyon, Pierre-Bénite, France; 9 INSERM U1060, Laboratoire CarMeN (cardiovasculaire, métabolisme, diabétologie et nutrition), Université Claude Bernard Lyon 1, Pierre-Bénite, France

**Keywords:** Vulnerable, Nutrition, Cancer, Colorectal tumor, Stepped wedge

## Abstract

**Background:**

Undernutrition prior to major abdominal surgery is frequent and increases morbidity and mortality, especially in older patients. The management of undernutrition reduces postoperative complications. Nutritional management should be a priority in patient care during the preoperative period. However undernutrition is rarely detected and the guidelines are infrequently followed. Preoperative undernutrition screening should allow a better implementation of the guidelines.

**Methods/design:**

The ANC (*“Age Nutrition Chirurgie”*) study is an interventional, comparative, prospective, multicenter, randomized protocol based on the stepped wedge trial design. For the intervention, the surgeon will inform the patient of the establishment of a systematic preoperative geriatric assessment that will allow the preoperative diagnosis of the nutritional status and the implementation of an adjusted nutritional support in accordance with the nutritional guidelines. The primary outcome measure is to determine the impact of the geriatric intervention on the level of perioperative nutritional management, in accordance with the current European guidelines. The implementation of the intervention in the five participating centers will be rolled-out sequentially over six time periods (every six months). Investigators must recommend that all patients aged 70 years or over and who are consulting for a surgery for a colorectal cancer should consider participating in this study.

**Discussion:**

The ANC study is based on an original methodology, the stepped wedge trial design, which is appropriate for evaluating the implementation of a geriatric and nutritional assessment during the perioperative period. We describe the purpose of this geriatric intervention, which is expected to apply the ESPEN and SFNEP recommendations through the establishment of an undernutrition screening and a management program for patients with cancer. This intervention should allow a decrease in patient morbidity and mortality due to undernutrition.

**Trial registration:**

This study is registered in ClinicalTrials.gov NCT02084524 on March 11, 2014 (retrospectively registered).

## Background

Colorectal cancer has the third highest incidence of cancer and the second highest mortality. The incidence and mortality curves significantly increase after the age of 55 years, with patients over 70 years old representing 60.5% of the incidence cases and 75.2% of cancer deaths [1, 2].

While the rates of perioperative morbidity and mortality have been drastically reduced in older patients due to the use of better anesthetic and post-surgical intensive care techniques [3, 4], age remains associated with a decrease in overall survival. The main mortality risk factors have recently been evaluated using a multivaried analysis and this predictive model has been prospectively validated on a test sample population [5]. The authors confirmed that the primary mortality risk factor is age: the risk is 1.8-fold higher between 80 and 85 years while beyond 90 years it is almost 3-fold higher. The increased rate of mortality in older patients compared to younger ones appears to be limited to the early postoperative period, as the disease specific survival ratio after this time is similar to that expected from the age-matched population as a whole [6]. The condition of the perioperative care therefore represents a priority for the improvement of colorectal cancer prognosis in older patients [7–11].

Beyond all the factors that affect the postoperative morbidity and mortality, the nutritional state of the patient is the most important [12]. Undernutrition significantly increases postoperative morbidity-mortality and is responsible for an increase in infectious and non-infectious postoperative complications and in the toxicity linked to the chemotherapy or radiotherapy treatment, leading to a prolongation of patient hospitalization and to an alteration of their quality of life [13].

**Fig. 1:**
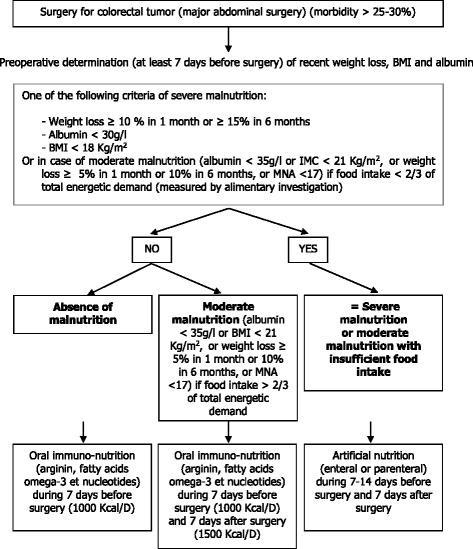
Recommendations and decision algorithm for the establishment of a nutritional support, according to the current recommendations of the European Society for Clinical Nutrition and Metabolism (ESPEN) and of the French Society of Clinical Nutrition and Metabolism (SFNEP)

In the case of colorectal cancer it has been shown that a weight loss greater than 10% prior to surgery [14] and undernutrition [15] are mortality risk factors. Since the rate of undernutrition is high in older hospitalized patients (up to 50%) [16–18], a nutritional assessment with undernutrition screening should be performed systematically before surgery in these subjects. The European guidelines, based on the results of many clinical studies (summary in Fig. 1), recommend undernutrition screening and routine nutritional care before, during, and after cancer surgery [3, 4, 19–27]. However, such guidelines with high levels of evidence are rarely followed [28, 29]. An unpublished pilot study that we have recently carried out showed that, in older patients hospitalized for colorectal cancer surgery, undernutrition was screened in less than 30% of cases and was not exhaustive. Perioperative nutritional care did not conform to the guidelines for over 95% of those patients undergoing endorectal surgery. Such insufficiency in patient screening and care could either be due to a lack of awareness of this health issue, a lack of interest in nutritional measurements by the medical staff, or by a lack of the necessary knowledge and time to rigorously apply the screening and nutritional care. The French geriatric pathway includes the Geriatric Team (GT), whose expertise is to ensure appropriate care and to improve the management of elderly patients. Our hypothesis is that the systematic intervention of the GT will lead to a better knowledge and respect of the perioperative nutritional care recommendations. This intervention will provide training in good practices, improve undernutrition screening in accordance with the guidelines, improve geriatric and nutritional management in surgery departments. It will also help to promote local paramedic referents via repeated training throughout the length of the study. Several training meetings will improve the identification and care of undernourished patients.

**Fig. 2:**
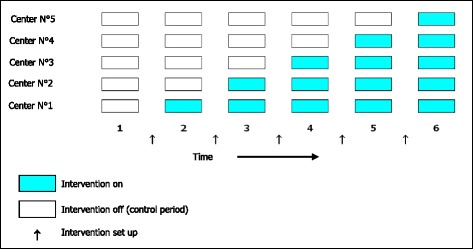
Schematic representation of the step wedge design of the ANC study: each line represents a center (*n* = 5). Each square represents a time unit (6 months period); white squares: control units (intervention is not yet implemented); blue squares: intervention units (intervention is implemented)

**Fig. 3:**
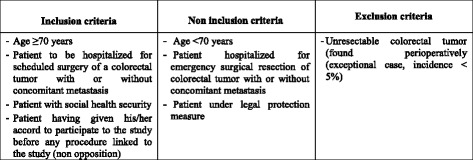
Summary of inclusion, non inclusion and exclusion criteria for the ANC study

**Fig. 4:**
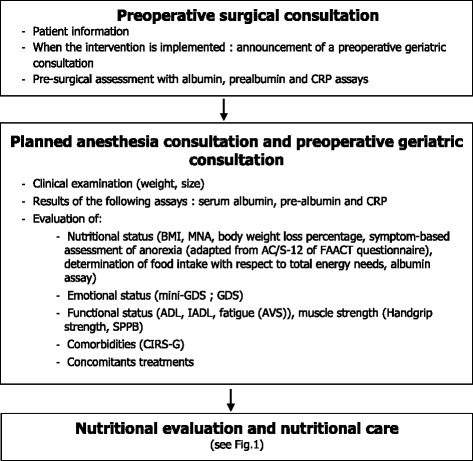
ANC study flow chart

## Methods/design

### Study design

The ANC protocol is an interventional, comparative, prospective, multicenter, randomized protocol based on the stepped wedge trial design [30, 31], which is appropriate for allowing the implementation of a nutritional intervention in older patients by a Geriatric Team (GT) within all participating services. This design involves the sequential rollout of an intervention in centers over several time periods. The order in which the different centers will receive the intervention will be determined at random. The implementation of the intervention in the five participating centers will be rolled-out sequentially over six time periods (every six months), in such a way that each center will initially belong to the “control” arm (without GT intervention) and then to the “intervention” arm (with GT intervention) (Fig. 2). Depending on the center, the pre-intervention phase, as well as the intervention phase, will last from 6 to 30 months. Thus each center will also act as its own control, which will reduce risk of bias and will allow comparison between each support modality (interventional compared to control) and in each group to analyze the effect of time on the effectiveness of an intervention and its learning. The study design will also avoid contamination between patients.

### Setting

The ANC protocol will take place in surgical wards of different French hospitals. Each hospital has its own GT composed of geriatricians and nurses. All the health professionals in the participating surgical wards will be involved in the conduct of the multidisciplinary prevention program (i.e. surgeons, anesthetist, nurses, and nurses assistants).

### Recruitment of patients

Patients eligible for the ANC study will be all subjects over 70 years old admitted for scheduled surgery of a colorectal tumor with or without concomitant metastasis and having given their informed consent to participate in the study. Patients with an emergency hospitalization for colorectal tumor surgery will not be included. (Fig. 3). Patients who are not included will receive the usual care in accordance with the current practices of the unit.

### ANC program

1. Pre-interventional phase
  An observational phase will precede the GT intervention. During this period, patients meeting the inclusion criteria will be included and followed by the digestive surgery unit in accordance with that unit’s current practices. Five patients will be included in each center per six months period. This phase will allow the usual practices of nutritional care in each surgical unit to be appraised.
2. Intervention
  The intervention provided by the GT will consist of a proposed training which will allow the surgical ward health professionals to follow the nutritional care recommendations and participate in the preoperative nutritional screening. Furthermore, the GT will propose, upon request from the surgery unit, a systematic implementation of preoperative geriatric assessment leading to a care in geriatric ward if necessary and the identification and training of local paramedical referents.
  - ***Training of surgical ward staff***
    The objective is to teach health professionals in order to follow the recommendations of the French National Health Authority (“Haute Autorité de Santé, HAS”), that are issued from the recommendations concerning perioperative nutritional care [19, 20], through the diffusion of informative documents and the organization of informative meetings.
  - ***Systematic implementation of preoperative geriatric assessment***
    A geriatric assessment is planned at least seven days before surgery. It will be performed by the geriatrician of the GT with four objectives:
    1. Assess the patient’s history, comorbidities and nutritional status:
      Nutritional assessment will be performed according HAS recommendation [1]:
      Notification of a body weight loss ≥5% (10% for severe cases) in one month,
      And/or Body weight loss ≥10% (15% for severe cases) in six months,
      Calculation of BMI <21 Kg/m^2^ (18 Kg/m^2^ for severe cases),
      Results of Albumin <35 g/l (<30 g/l for severe cases),
      Global Mini Nutritional Assessment (MNA) <17/30.
      In addition, each patient will be submitted to a dietetic interview in order to evaluate his daily food intake.
      Evans criteria for cachexia diagnostic will be recorded, defined by the association of a body weight loss of ≥5% in 12 months (or BMI <20 Kg/m^2^) associated with at least three of the following criteria [32]:
      Decrease of muscle strength (hand grip strength <20 Kg for women and <30 Kg for men) [33],
      Analogical Visual Scale (AVS) Fatigue score ≤3 [33],
      Anorexic syndrome: defined as a decrease in appetite, responsible of a reduction of total calorie intake (<20 Kcal/Kg of body weight/day) or a 2/3 reduction of requested food intake evaluated between 14 and 7 days before surgery or a score ≤23.5 on the anorexic scale (adapted from the questionnaire AC/S12 of FAACT) [34],
      Decrease of the Lean Mass Index evaluated by the GT between 14 and 7 days before surgery as a calf circumference <31 cm (Mini Nutritional Assessment, MNA; [35]),
      Abnormal biochemical values (1 of the 3): CRP >5.0 mg/l, hemoglobin <12 g/dl (anemia), Serum-albumin <32 g/l.
    2. and perform a complete clinical examination and a standardized geriatric assessment focusing on:
      Measurement of weight (Kg) and height (m)
      Nutritional biologic markers will be systematically collected: albuminemia, pre-albuminemia, C-reactive protein, hemoglobin.
      Assessment of functional status using the Short Physical Performance Battery (SPPB) [36], the Activity Daily Life (ADL, [37]) and the Instrumental Activity Daily Life (IADL, [38]).
      Assessment of emotional status using the Geriatric Depression Scale (GDS, [39])
      Assessment of co-morbidities (CIRS-G test)
    3. propose an adapted preoperative nutritional management according to the nutritional status of the patient using the decision tree for nutritional care [20];
    4. advise the surgery team of the nutritional recommendations to follow in the postoperative phase (Fig. 1).

### ANC study schedule

In each surgical department involved in the study, the surgeon will screen all patients for trial eligibility during a preoperative surgical consultation. Patients in the observational period will receive the usual care, in accordance with the current practices of the unit. In the interventional period, the surgeon will inform the patient of a systematic preoperative geriatric assessment.

For the implementation of the intervention, the GT assessment will be standardized for all centers. It will last approximately 90 min and will be carried out by the GT team, together with a dietician, in the surgery or anesthesia unit, depending on the practices of the center. A nutritional management plan, adapted to the nutritional status of the patient, will be prescribed and implemented following this assessment, in accordance with the current recommendations and decision algorithm (Fig. 4).

### Outcome and measurement

The primary outcome measure is to determine the impact of the geriatric intervention on the level of perioperative nutritional management, in accordance with the current European guidelines, of patients aged 70 years old or above who are undergoing surgery for colorectal cancer. This will be accomplished by evaluating the percentage of patients having received a nutritional screening i.e. measurement of body weight and size, calculation of the body mass index (BMI), evaluation of a recent body weight loss and an albumin assay at least 7 days before surgery, and having received oral immunonutrition during 7 days before surgery (if absence of malnutrition) or immunonutrition during 7 days before and 7 days after surgery (if moderate malnutrition) or artificial nutrition during 7–14 days before surgery and 7 days after surgery.

The secondary outcomes are:

1. The percentage of patients having received a nutritional screening, i.e. measurement of body weight and size, calculation of the body mass index (BMI), evaluation of a recent body weight loss and an albumin assay at least 7 days before surgery.
2. The percentage of patients having received oral immuno-nutrition
3. The percentage of patients with undernutrition, severe undernutrition and cachexia
4. The rate and type of postoperative complications, from grade I to V, according to the Common Terminology Criteria for Adverse Events v4.0 (CTCAE) of National Cancer Institute, occurring up to 30 days after surgery.
5. The number of actual oral nutritional supplements intakes with respect to planned ones (noted in the patient diary given at the preoperative geriatric assessment).
6. The comparison of functionality scores obtained on the ADL and IADL, evaluated by the GT between the beginning (between 14 and 7 days before surgery) versus the end (after surgery, the day before or the day of discharge from the surgical ward) of the hospitalization period.
7. The emotional state of the patient evaluated with the mini-GDS (score on 4) and the GDS 15 (if mini GDS score >2).
8. The number of concomitant treatments (number of drug specialties between 0 and 3, between 3 and 6, over 6) during the hospitalization period.

### Data collection

The data for the CRF (Case Report Form) will be collected and recorded by the Clinical Research Assistant (CRA) from the patients’ medical files.

### Sample size

The sample size was defined using the appropriate formula for stepped wedge studies [30]. It was calculated by considering the most unfavorable situation that would result in a statistical difference, i.e 5% of pre- and post-operatively nutritional management in accordance with the current European guidelines in the control group vs. 20% in the intervention group. The significance threshold was fixed at 5%. The number of centers is 5 and the number of time period has been set at 6 to obtain optimum power. The coefficient of variation should be between 0.15 and 0.40, we set at 0.40 to be in the most pessimistic conditions. Five patients per center/step will be required for a power of 84%. This corresponds to a total of 150 patients (30 for each center).

### Statistical analysis

In order to ensure the objectivity of the results, the statistical analysis for this study will be performed by an independent statistician at the end of the follow-up period of the last patient included. The data will be analyzed using the SAS 9.1 software (Cary Inc., North Carolina, USA). No intermediate analysis is planned. Confidence intervals will be calculated according to an alpha risk of 5% and the statistical test results will be given a 5% threshold.

Univariate descriptive analysis will be used. The patients’ characteristics will be described and compared between the subsequent time points of the centers in which the intervention has started (intervention group) and those in which it has not (control group). The analysis will concern the patients’ demographic (age, sex) and clinical (antecedents and associated illnesses, surgical indication, etc.) characteristics, as well as the type and location of the intervention that has been performed. The comparisons will be evaluated using the Student *t*-test or the corresponding nonparametric test for the quantitative variables (average comparisons) and the Chi-Square or the Fisher’s test for the qualitative variables (percentage comparisons).

Concerning the primary endpoint analysis, the nutritional management of pre- and postoperative rate according with the recommendations will be compared before and after implementation of the intervention. This comparison will be performed by a logistic regression integrating time and the group (intervention / control) and an adjustment on the group (GLMM = generalized linear mixed model).

The support levels before and after implementation of the intervention will be presented on a gross basis and compared using a Chi square test. A graphical analysis will view the support levels before and after implementation of the intervention for each of the centers.

Concerning the analysis of secondary outcomes:

The percentage of subjects who underwent screening and the percentage of patients who received a care prescription will also be compared using a GLMM.

Globally (without taking into account the intervention) will be described:

* malnourished subjects, malnourished subjects who could not get a prescription for nutritional management, severe malnourished patients, cachectic subjects among all subjects who underwent screening (percentage and 95% confidence interval)
* the emotional score, the mini GDS on subjects who received screening, the number of concomitant treatments and specialties, the number of food intake on the number of prescribed taken, the average change in IADL score (mean, standard deviation, median, minimum, maximum).

The percentage of complications will also be compared between subjects who received nutritional support and those who don’t have benefited of this support by a Chi square test.

### Ethical considerations

Investigators must recommend that all patients aged 70 years old or over, consulting for a surgery for colorectal cancer, should consider participating in this study. There should be no pre-selection of the patients by the surgeon. The participating patients will give their consent after being told about the study. Their decision to accept or refuse participation will not affect their medical and nursing care.

### Trial status

The enrolment of patients has begun, but data cleaning or analysis had not yet begun as of this article’s submission.

## Discussion

### Discussion of the study design

The ANC study is based on an original methodology, the stepped wedge trial design which differs from the parallel and cross-over designs and presents several advantages. Due to the type of the intervention, the risk of contamination would have been too high with a one to one randomization, hence the need for clusters. The stepped wedge design may require fewer centers than a parallel group design to ensure similar group comparability [30, 31, 40–42].

This type of study design presents benefits with regard to implementation: the sequential introduction of the intervention in all the centers does not disadvantage any particular center. The implementation will be easier and it can facilitate the organization of the study in routine practice [43].

Moreover, the risk of center bias is minimized because each center is its own control and receives the intervention. Therefore, the impact of the intervention can be estimated from both between and within center comparisons [30, 31, 40, 44].

This design also allows an evaluation of the effects of time period on protocol effectiveness (following the temporal evolution of the application of the recommendations in the centers). Centers will differ in the time spent on the intervention. Centers that switch at the first time period will be more experienced with the ANC intervention than centers that switch at last time period [44].

Finally, the stepped wedge design allows each participating center to benefit from the ANC intervention. This opportunity is a motivational factor for taking part in this study. In accordance with good ethical practice, the intervention is expected to do more good than harm.

### Discussion of the intervention

The principal aim of this study is to increase the implementation of the recommendations for perioperative nutritional care for colorectal cancer in elderly patients [19, 20]. In spite of their importance, such recommendations are rarely followed [28, 29]. Our suggestion is that the intervention of the geriatric team (GT) in the perioperative period will help to reduce post-surgical complications and the associated mortality – morbidity. Consequently, the application of the ANC protocol could also lead to a reduction in the length of the post-surgical hospital stay. The ANC study will also develop or strengthen the collaboration between surgical wards and the GT, and the application of actual nutritional recommendations should improve during and after the study.

The introduction of GT in the majority of multidisciplinary hospitals should improve the level of awareness and knowledge of undernutrition-related problems in elderly patients hospitalized for surgery through the training provided by the GT in the wards. It should allow a comprehensive and systematic screening for undernutrition in elderly patients hospitalized for colorectal cancer based on the knowledge of patient weight loss, body mass index, albumin concentration and MNA. It could also promote the application of the good practice recommendations (HAS, ESPEN, SFNEP) and help to develop collaboration and communication among health professionals. Furthermore, in case of severe undernutrition, a specific treatment including artificial nutrition should be discussed in accordance with the recommendations. It is also important to take care of patients with moderate undernutrition in order to prevent the establishment of cachexia.

In summary, the modification of practices induced by the ANC study should allow a decrease in undernutrition-related morbidity and mortality in patients, as well as a decrease in the cost of hospitalization (shortening of the stay and reduction of complications).

Finally, after the completion of the ANC study, the maintenance of the implemented intervention (awareness, collaboration, support, nutritional status diagnosis and nutritional care) and the dissemination of the results will be proposed so that this type of intervention can be extended to other wards and to other types of surgery.

## Abbreviations

ADL: Activity daily life
ANC: *Age Nutrition Chirurgie*
AVS: Analogical visual scale
BMI: Body mass index
GDS: Geriatric depression scale
GT: Geriatric team
IADL: Instrumental activity daily life
MNA: Mini nutritional assessment
SPPB: Short physical performance battery
